# Isolation and characterization of plant plasma membrane P-type H^+^-ATPase dimers

**DOI:** 10.1038/s41598-026-70970-z

**Published:** 2026-09-15

**Authors:** Ekaterina Malysenko, Thomas Günther Pomorski, Bo Højen Justesen

**Affiliations:** 1 Department of Molecular Biochemistry, Faculty of Chemistry and Biochemistry, Ruhr University Bochum, 44780 Bochum, Germany; 2 Department of Plant and Environmental Sciences, University of Copenhagen, 1871 Frederiksberg, Denmark

**Keywords:** H^+^-ATPase, Oligomers, Dimers, Membrane reconstitution, Proton pumping, SEC-MALS, Biochemistry, Biophysics, Biotechnology

## Abstract

Plant H⁺-ATPases are activated through tightly regulated hexameric assembly, relieving autoinhibition via displacement of a regulatory domain during the oligomerization process. Sub-hexameric assembly states also exist but their role in activity regulation remains elusive. To characterize sub-hexameric plant H⁺-ATPases, we isolated homo-oligomers of the H⁺-ATPases AHA2 and determined their sizes with size exclusion chromatography multi-angle light scattering. AHA2 predominantly presented as a monomer but also assembled into stable dimers and larger complexes. Activity assays on solubilized and liposome-reconstituted AHA2 monomers and dimers revealed that dimerization did not affect enzymatic activity, suggesting that AHA2 dimerization does not represent a separate regulatory pathway. Instead, AHA2 dimers may serve as reservoirs for rapid hexamer assembly, facilitating the dynamic regulation of protein activity.

**Supplementary Information:**

The online version contains supplementary material available at https://doi.org/10.1038/s41598-026-70970-z.

## Introduction

The P3-type ATPase subfamily consists of plasma membrane H⁺-ATPases that constitute the driving force behind electrochemical membrane gradients in plant and fungal cells^1–4^. These proton pumps are organized into ten transmembrane helices and three catalytic cytosolic domains^5–7^. Adenosine triphosphate (ATP) hydrolysis at the cytosolic domains enables a cycle of auto-phosphorylation and dephosphorylation that leads to conformational changes and proton transport^8–10^. The transport cycle can be influenced by autoinhibition exerted by regulatory domains located on the N- and C-termini^11–14^. Through this regulatory mechanism, the proton pumps can switch between an autoinhibited state with basal activity and an activated state upon kinase-mediated phosphorylation of the C-terminal regulatory domain^15,16^. Activation and hexamer formation appear to be linked in plant proton pumps. In the proposed mechanism for plant proton pump hexamer formation, pre-formed dimers are phosphorylated at the penultimate threonine of the C-terminus^17,18^. This phosphorylation stabilizes the binding of a single phosphorylated C-terminus to a 14-3-3 protein dimer^13,18–21^. Three of the H^+^-ATPase/14-3-3 hetero-complexes then arrange into a complex with six proton pumps and six 14-3-3 proteins^17,18^. Release of the autoinhibitory C-terminus results in activation of the proton pumps, and the formation of hexamers therefore appears to be associated with a marked increase in activity^17,19–22^. The propensity of plant proton pumps to complex with 14-3-3 proteins and the stability of the resulting complex have been shown to be dependent on the plant species and isoform^17,23–25^. Other than hexamers, structural characterizations have also revealed sub-hexameric assembly states of plant proton pumps, such as monomers, dimers and trimers, both in membranes^17,26^ and after purification^25,27–29^. Plant proton pump monomers display activity, indicating that hexamer formation is not required for proton transport^27^. Nevertheless, it is not known how monomers and sub-hexameric oligomers may contribute to plant H^+^-ATPase function in vivo. Even though the current model proposes that activation via release of the autoinhibitory domains occurs before hexamers are assembled, there are indications that the activated state is mostly populated by hexamers. Both threonine phosphorylation and 14-3-3 protein binding seem enhanced within proton pump hexamers compared to dimers^17^. This could indicate that dimers constitute a resting state of plant proton pumps in the plasma membrane, while activated dimers rapidly assemble into hexamers. It is unclear whether proton pump monomers are also able to form oligomers in response to phosphorylation and 14-3-3 protein binding. Further characterizations of plant proton pump dimers and monomers require the separation of the different assembly states, which can only be achieved after protein purification. In this study, monomers and dimers of the proton pump *Arabidopsis thaliana* plasma membrane H^+^-ATPase isoform 2 (AHA2) were isolated after detergent-based purification. Size exclusion chromatography coupled to multi-angle light scattering (SEC-MALS) was used to separate purified AHA2 oligomers and to determine the molecular weights of the eluting complexes. For functional characterization, solubilized and liposome-reconstituted AHA2 monomers and dimers were probed in activity assays to investigate the role of AHA2 dimerization in AHA2 activity modulation.

## Results

### Isolation and characterization of stable AHA2 oligomers

**Fig. 1:**
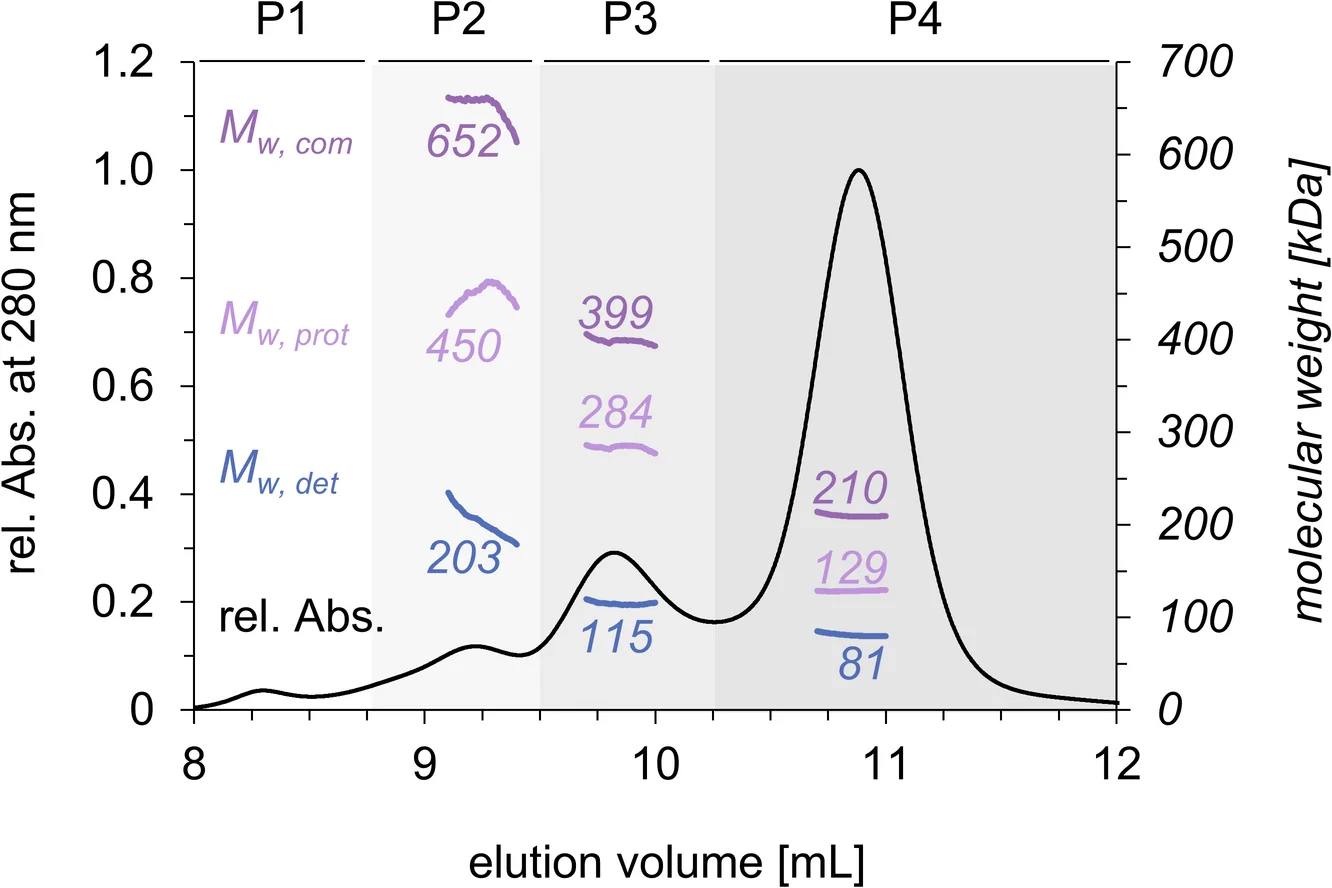
Size characterization of AHA2 oligomers. DDM-solubilized AHA2 eluted in four absorbance peaks (P1-4), with P1 corresponding to the dead volume. Molecular weights of the protein component (M_w, prot_), protein–detergent complex (M_w, com),_ and detergent micelle (M_w, det_), were calculated from SEC-MALS data and averaged over defined elution ranges with the relevant data points shown as curves. AHA2 oligomeric states were determined by comparing M_w, prot_ with the AHA2 monomer molecular weight (127 kDa). Peak tailing and M_w_ variability in P2 indicated heterodispersity. Two additional analyses of independent purifications are shown in the Supporting Information (Supplementary Fig. S2; *n* = 3).

Previous reports indicated that AHA2 forms homo-oligomeric complexes in membranes that can be recovered after purification^26–29^. Consistent with this, detergent-solubilized and affinity-purified AHA2 (Supplementary Fig. S1) displayed multiple absorbance peaks (P1-4) in size exclusion chromatography (SEC; Fig. 1, Supplementary Fig. S2). Analysis of the protein content in the eluted fractions revealed that AHA2 was the only detectable protein, confirming that the multipeak elution profile corresponds to different AHA2 homo-oligomers (Supplementary Fig. S3). SEC-MALS enabled molecular weight determination of the eluting complexes (Fig. 1, Supplementary Fig. S2). As purified AHA2 was solubilized in DDM micelles, the size determination was subdivided into three categories: the total size of AHA2 and the detergent micelle (M_w, com_), the size of only the AHA2 component (M_w, prot_) and the micelle size (M_w, det_). For homo-oligomers, M_w, prot_ should constitute multiples of the molecular weight of AHA2 monomers (127 kDa). Accordingly, AHA2 eluted in P4 corresponded to monomeric AHA2, while proteins eluted in P3 exhibited a molecular weight consistent with AHA2 dimers. P2 exhibited increased peak tailing and high variability in molecular weight, suggesting the presence of multiple co-eluted species, with the resulting AHA2 complex size in-between AHA2 trimers and tetramers. When particles of different sizes and compositions display overlapping elution, molecular weight determination becomes less reliable. As AHA2 trimers have previously been described^26^, the complexes eluting in P2 were presumed to mostly represent trimeric AHA2. A fraction of AHA2 eluted in the void volume (P1), indicating complex sizes beyond the molecular weight exclusion limit of 600 kDa. Yet, the analysis of total AHA2 using a SEC column with a higher molecular weight exclusion limit ruled out the presence of stable hexamers (Supplementary Fig. S2). The detergent molecular weight in P4 resembled the size of an empty DDM micelle (66 kDa)^30^, but increased in P3 and P2, suggesting an increase in micelle size with increasing AHA2 complex size. However, M_w, det_ may also include other compounds besides detergent that are not detected via UV absorbance. Therefore, an increase in M_w, det_ could also imply the co-purification of other compounds, such as lipids.

Detergent solubilization of membrane proteins can result in atypical complex formation: They may aggregate into artificial complexes, or pre-existing oligomers may be disrupted^31–33^. Several observations argue against these outcomes for AHA2. First, the identified complexes remained stable even when the detergent concentration was increased sixfold to disrupt non-specific protein interactions (Supplementary Fig. S4). Second, isolated monomer fractions did not redistribute into a multi-peak chromatogram (Supplementary Fig. S5). Moreover, isolated fractions enriched in higher-molecular AHA2 complexes did not show regeneration of AHA2 monomers over time, indicating that solubilized AHA2 complexes exist outside of a dynamic redistribution equilibrium (Supplementary Fig. S6). Third, diluted AHA2 trimers (14–67 µg mL^−1^) isolated from SEC remained detectable, demonstrating that the dilution did not affect the observed distribution of AHA2 oligomers (Supplementary Fig. S6). Having isolated stable fractions enriched in AHA2 monomers and dimers, respectively, these were used for activity characterizations.

### Activity of detergent-solubilized AHA2

**Fig. 2:**
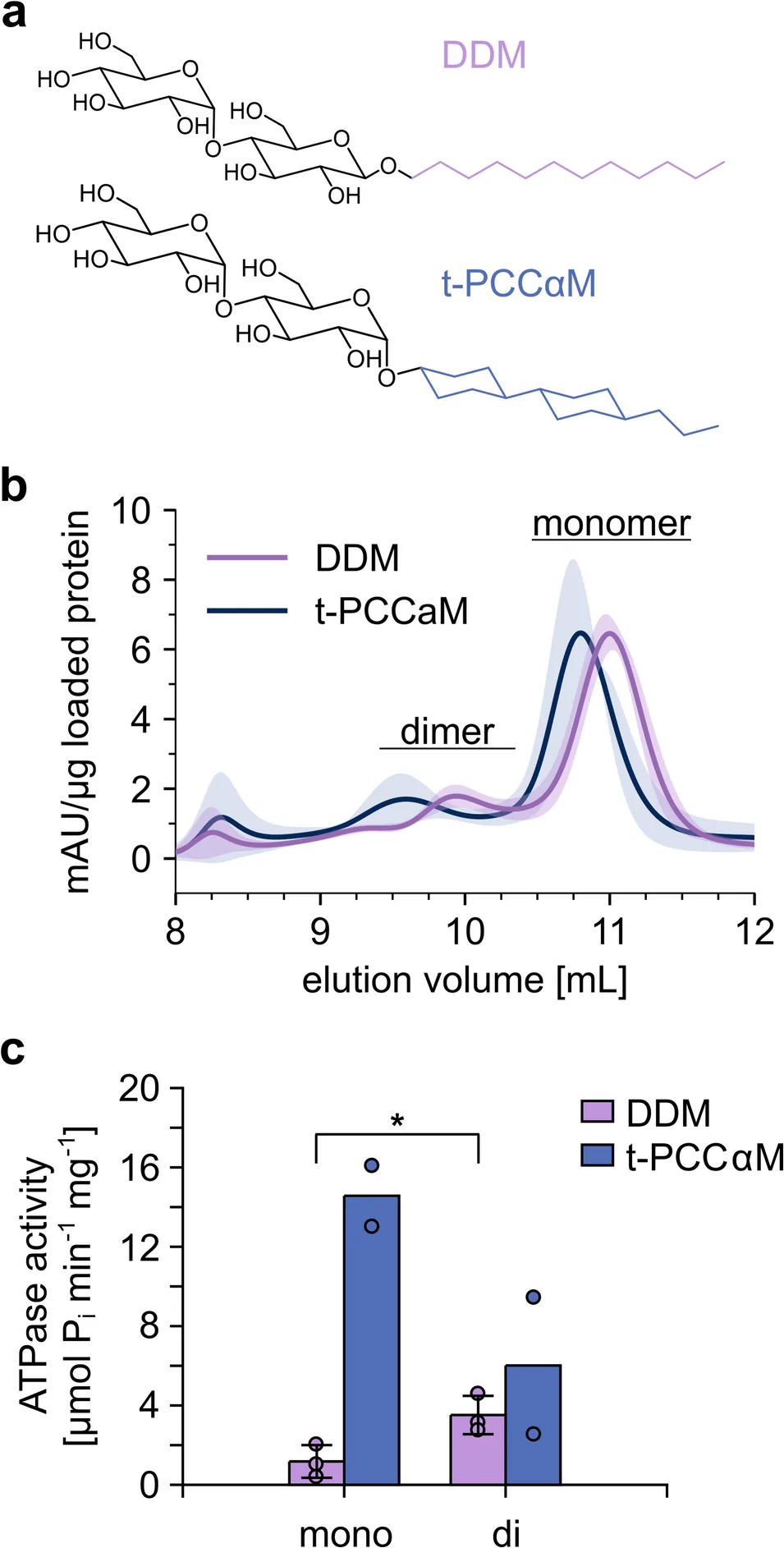
ATPase activity of detergent-solubilized AHA2. (**a**) AHA2 was solubilized and purified in DDM or t-PCCαM. (**b**) Size exclusion chromatography of AHA2 in both detergents showed similar elution profiles, with monomer and dimer peaks slightly shifted to lower volumes in t-PCCαM. Chromatograms are averages of independent AHA2 purifications, with ribbons representing ± S.D. (DDM *n* = 4, t-PCCαM *n* = 2). (**c**) Fractions corresponding to AHA2 monomers (mono) and dimers (di) were selected based on absorbance maxima. Detergent-solubilized AHA2 monomer and dimer ATPase activity was measured in presence of DOPC. Values are means from independent purifications, while error bars are ± S.D. (DDM *n* = 3, t-PCCαM *n* = 2). Significance was evaluated using Welch’s test (*, *p* < 0.05).

While proton transport cannot be measured on solubilized AHA2 due to the lack of an enclosed membrane compartment, ATPase activity can be probed. However, AHA2 ATPase activity can be influenced by detergents^34^. To evaluate potential detergent-specific effects, AHA2 was purified using the maltoside-based detergents DDM and t-PCCαM (Fig. 2a), which exhibited similar solubilization efficiencies (Supplementary Fig. S7). Independent of the detergent, size exclusion of AHA2 produced similar elution profiles, however, t-PCCαM-solubilized AHA2 eluted earlier, which is consistent with an apparent increase in molecular weight (Fig. 2b). This points towards larger t-PCCαM micelles, either due to more detergent molecules required for an AHA2-enveloping micelle, as empty micelles are similar in size to DDM micelles^30^, or due to co-purified lipids. Relative amounts of AHA2 monomers and dimers appeared similar in both detergents, but trimers were not detected in t-PCCαM-purified samples, as trimer elution may have been shifted into the void volume because of the molecular weight increase. After relipidation with DOPC, the specific ATPase activity of purified AHA2 monomers and dimers was measured (Fig. 2c). Dimer ATPase activity appeared unaffected by the detergent choice, but monomer activity differed. With DDM, the dimer activity was threefold higher than the monomer activity. In contrast, the activity of t-PCCαM-solubilized monomers was much increased and fivefold higher than the dimer activity. These differential effects of detergents on monomers and dimers indicated that solubilized AHA2 is not suitable for comparing oligomer-specific ATPase activities.

### Characterization of reconstituted AHA2

**Fig. 3:**
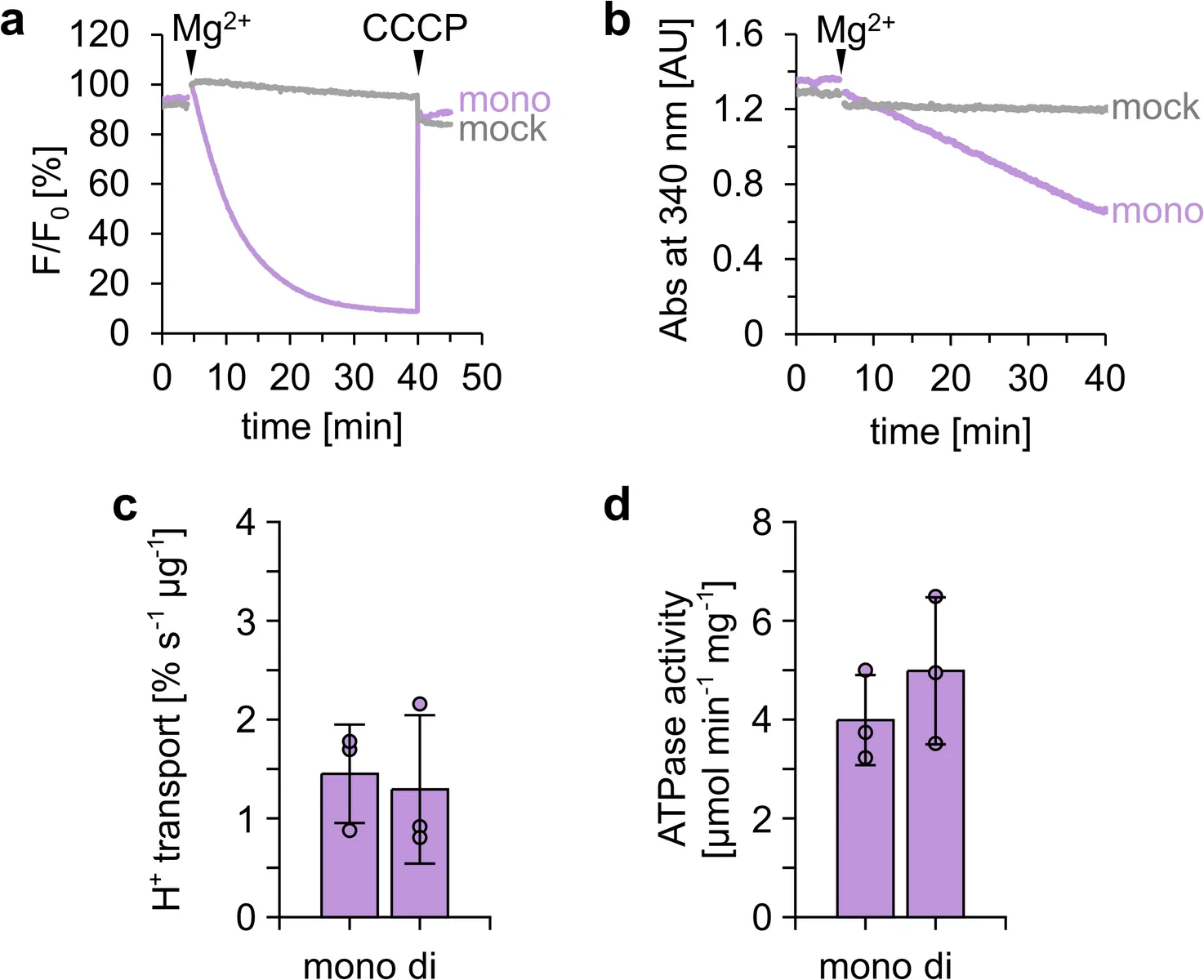
Activity of AHA2 proteoliposomes. DDM-solubilized AHA2 monomers (mono) and dimers (di) were reconstituted into lecithin liposomes, and proton pumping and ATPase activities were measured. (**a**) Proton pumping in proteoliposomes was monitored via ACMA fluorescence normalized to initial fluorescence (F/F_0_). Monomer proteoliposomes showed Mg²⁺-ATP-dependent fluorescence quenching, absent in mock liposomes; CCCP addition reversed quenching. (**b**) ATPase activity was determined using an NADH-dependent enzymatic assay coupled to ATP hydrolysis. (**c**, **d**) Proton transport rates and specific ATPase activities for reconstituted AHA2 monomers and dimers (*n* = 3, independent purifications).

To eliminate possible detergent effects, DDM-purified AHA2 monomers and dimers were reconstituted in lecithin liposomes, enabling measurements of both H^+^-transport and ATPase activity. H^+^-transport was determined from the initial rate of ACMA fluorescence decrease, reflecting proton accumulation within the proteoliposomes after providing Mg^2+^-ATP (Fig. 3a). ATPase activity was measured in an enzymatic assay that coupled ATP hydrolysis to NADH consumption by calculating the rate of NADH absorbance decrease (Fig. 3b). Activities were normalized to the amount of AHA2 in each proteoliposome sample as determined by SDS-PAGE using a BSA standard series (Supplementary Fig. S8). Functional analysis revealed that reconstituted AHA2 monomers and dimers had similar H^+^-transport and ATP hydrolysis rates (Fig. 3c,d), contrary to the observations for detergent-solubilized AHA2. Under DDM-solubilized conditions, AHA2 dimers displayed higher ATPase activity than monomers following relipidation with either DOPC (Fig. 2c) or lecithin (Supplementary Fig. S9), indicating that the lipid composition did not influence the activities of monomers and dimers measured in the proteoliposomes. Furthermore, the preservation of distinctly different oligomer compositions after reconstitution of AHA2 monomer and dimer fractions was confirmed by detergent solubilization of proteoliposomes followed by SEC analysis (Supplementary Fig. S10). These results suggest that the formation and dissociation of dimers do not constitute a regulatory mechanism for the plant proton pump AHA2.

## Discussion

The homo-oligomerization of plant proton pumps into hexamers is a mechanistically intricate phenomenon involving tight regulation in the form of post-translational modifications and protein interactions necessary to form the complex. Structural evidence from heterologous expression studies suggests that the plant proton pump AHA2 may exist as monomers, dimers, and trimers in the membrane^25–28^. These findings align with our observations of AHA2 oligomeric states purified from yeast membranes following heterologous expression of full-length AHA2. Size determination by SEC-MALS convincingly revealed AHA2 monomers and dimers in the purified preparations (Fig. 1). As AHA2 trimers have previously been described, the peak P2 in the size exclusion chromatogram may represent a trimeric assembly of AHA2 monomers. This reveals a limitation of the present analysis, wherein SEC-MALS confidently identifies AHA2 monomers and dimers, while the assignment of larger oligomer species remains uncertain. Moreover, the structural arrangement of AHA2 protomers within these assemblies cannot be inferred from SEC-MALS alone. Additional structural approaches will be required to determine whether these species represent defined intermediates in native hexamer formation.

Under the purification conditions used here, no AHA2 hexamers were isolated. Even though plant proton pumps have previously been shown to form complexes with yeast 14-3-3 proteins^22^, 14-3-3 proteins were not detected in the SEC fractions analyzed in this study (Supplementary Fig. S3). Reportedly, these complexes of plant proton pumps and 14-3-3 proteins appear inherently unstable in the absence of stabilizers such as the fungal toxin fusicoccin^17,24,35^. SDS-PAGE analysis revealed no co-elution of any other proteins with the AHA2 dimer and trimer fractions, indicating that the purified AHA2 oligomers can assemble independently of associated stabilizing proteins. Beyond proteins, lipids have also been implicated in stabilizing protein complexes^33,36^. Some indication for the co-purification of lipids with AHA2 oligomers was found in SEC-MALS analysis: The apparent molecular weight of non-protein components, here attributed to the detergent micelle, scaled with increasing AHA2 complex size. However, the analysis cannot distinguish between detergent molecules and other substances undetectable via UV absorbance, including lipids. Therefore, the observed increase in non-protein mass may not simply reflect swelling of detergent micelles to accommodate larger protein complexes but could also indicate co-purified lipids. The contribution of lipids to AHA2 oligomer stabilization remains to be investigated in future studies. The isolated AHA2 oligomers were found suitable to investigate whether dimerization is instrumental in activity regulation. For detergent-solubilized AHA2, different specific ATPase activities were measured between monomers and dimers, however, these differences depended on the detergent used for purification. DDM and t-PCCαM have previously been found to have different impacts on the stability and activity of proteins^30,37^. Here, we demonstrated that these detergents impact AHA2 monomers and dimers to different extents, likely due to detergent-binding sites revealed or obstructed depending on the assembly state of AHA2. Additionally, the observed differences in activity between monomers and dimers were below an order of magnitude, further challenging the idea that a monomer-dimer transition alone drives significant functional regulation. The primary known mechanism of AHA2 regulation is autoinhibition via the terminal regulatory domains, which has been reported to be compromised during purification in detergents, leading to partial activation of the protein^38^. Therefore, activity analysis of purified and reconstituted AHA2 monomers and dimers does not permit a complete evaluation on how oligomerization is involved in this canonical mode of activation. Nonetheless, regulation independent of autoinhibition is possible, e.g., activation by anionic lipids^39^. Our data indicates that dimerization does not represent such an alternative regulatory pathway, as reconstituted AHA2 monomers and dimers exhibited similar ATPase and proton pumping activities. Previous studies proposed that plant proton pump dimers serve as a reservoir of building blocks for activated hexamers^17,18^. Dimers are likely the assembly state that binds 14-3-3 dimers, a critical step in hexamer formation. Whether monomers would also be capable of this interaction has not been investigated, although it has been proposed that the 14-3-3 dimer can only bind one C-terminal regulatory domain of the proton pump dimer^17,18^. Conceivably, the binding of two pump monomers to a 14-3-3 dimer would result in a premature termination of hexamer formation, whereas proton pump dimers would offer another C-terminal domain to continue complex formation. For plant proton pumps, hexamer formation coincides with activation as the autoinhibitory domains are released and captured by the 14-3-3 scaffold proteins. A hexameric complex may provide special support of the activated state, as it has been proposed for fungal proton pumps based on structural data^40,41^. In summary, while AHA2 dimers may not constitute an independent mode of activity regulation for proton pump activity, they may contribute towards the release of canonical AHA2 autoinhibition.

## Methods

### Materials

Detergents *n*-octyl β-D-glucopyranoside (OG), *n*-Dodecyl β-D-maltoside (DDM) and 4-trans-(4-trans-propylcyclohexyl)-cyclohexyl α-maltoside (t-PCCαM) were obtained from Glycon (Luckenwalde, Germany). Protein purification was performed with Protino^®^ Ni-NTA agarose (Macherey-Nagel, Düren, Germany). The lipid 1,2-dioleoyl-*sn*-glycero-3-phosphocholine (DOPC) was purchased from Avanti Polar Lipids (Alabaster, AL, USA). Sephadex™ G50 fine was ordered from VWR Chemicals (Leuven, Belgium), Bio-Beads SM-2 resin was purchased from Bio-Rad (Hercules, CA, USA). Glycerol (≥ 99%) was acquired from Fisher Chemical (Loughborough, United Kingdom). All other chemicals were obtained from Sigma-Aldrich (Steinheim, Germany), unless otherwise indicated. Buffers and solutions for use on High Performance Liquid Chromatography (HPLC) equipment were filtered through 0.1 μm polyethersulfone bottle-top filters (Sarstedt, Nümbrecht, Germany).

### Preparation and solubilization of membranes

A plasmid based on the vector Yep-351^42^ with the coding sequence of AHA2 from *A. thaliana* was transformed into the *S. cerevisiae* strain RS-72 (*MATa*,* ade1-100*,* his4-519*,* leu2-3*,* 112*) for expression^43^. The plasmid encoded an N-terminally tagged AHA2 fusion protein with a MRGSH_6_-tag for affinity purification, a StrepII-tag, and a SNAP^®^-tag. The fusion protein was expressed under the promoter of the native yeast proton pump *PMA1*. The yeast strain RS-72 has the endogenous *PMA1* under the control of a galactose-induced *GAL1* promoter^43^. The yeast was transformed and cultured essentially as described previously^9^. Cultures were expanded in SGAH (2% w/v galactose, 0.7% w/v yeast nitrogen base without amino acids, 40 mg L^−1^ adenine, 20 mg L^−1^ histidine, 50 mM succinic acid-tris, pH 5.5) and switched to YPDA (1% w/v yeast extract, 2% w/v peptone, 2% w/v D-glucose, 40 mg L^−1^ adenine) for overexpression (130 rpm, 20 h, 30 °C). Cells were harvested by centrifugation (1400 × g, 7 min, 4 °C), lysed with glass beads in homogenization buffer (50 mM 2-(*N*-morpholino)ethanesulfonic acid (MES), 50 mM KCl, 26% v/v glycerol, 10 mM ethylenediaminetetraacetic acid (EDTA), 1 mM dithiothreitol (DTT), 0.3 mM phenylmethylsulfonyl fluoride (PMSF), 3 µg mL^−1^ pepstatin A (PepA), 1.2 mM ATP, pH 6.4 with KOH), and membranes were isolated by ultracentrifugation at 184,000 × g (50.2 Ti fixed-angle rotor, Optima XPN-80 ultracentrifuge; Beckman Coulter, Brea, CA, USA)^44^. Membrane pellets were resuspended in solubilization buffer (50 mM MES, 50 mM KCl, 20% v/v glycerol, 0.7 mM EDTA, 0.7 mM DTT, 0.2 mM PMSF, 2 µg mL^−1^ PepA, 2.17 mM ATP, pH 6.4 with KOH) and dounced using a glass homogenizer. Protein concentrations were determined by *DC*™ protein assay (Bio-Rad), and solubilization efficiency was assessed by detergent treatment (10 mg mL^−1^ membranes and 0–1% w/v detergent, incubated for 1 h at 4 °C) followed by ultracentrifugation (150,000 × g, 30 min, 4 °C) and SDS-PAGE/western blot analysis.

### SDS-PAGE, coomassie staining and western blotting

Proteins were separated on 10% SDS-PAGE gels using standard Tris-glycine-SDS buffer and PageRuler Plus Prestained Ladder (#26619, Thermo Fisher Scientific, Waltham, MA, USA). Proteins were either stained in-gel with colloidal Coomassie^45^ or transferred to 0.2 μm PVDF membranes (Bio-Rad). Membranes were blocked with 5% w/v milk in TBST (10 mM Tris, 150 mM NaCl, 0.1% v/v Tween 20, pH 8.0 with HCl) and probed with mouse anti-RGS·His_4_ (#34650, Qiagen, Hilden, Germany) primary and alkaline phosphatase-conjugated anti-mouse (#A4312, Sigma-Aldrich) secondary antibodies, both diluted 1:10,000. To assess unspecific secondary antibody binding, control membranes containing 5 µg of protein were processed in parallel by incubation with either primary antibody solution or TBST alone, followed by incubation with the secondary antibody solution. Detection was performed with 5-bromo-4-chloro-3-indolyl-phosphate/nitro blue tetrazolium chloride (Roche, Basel, Switzerland), and images were acquired on a ChemiDoc MP system (Bio-Rad). Band intensities were quantified with Image Lab version 6.0 build 7 (Bio-Rad).

### Protein purification

AHA2 purification was carried out according to Pedersen et al.^44^. Membranes were solubilized with 0.5% w/v DDM or t-PCCαM by inverting gently for 1 h at 4 °C and clarified by centrifugation (150,000 × g, 90 min, 4 °C). Supernatants were diluted 1:1 with wash buffer (50 mM MES, 500 mM KCl, 20% v/v glycerol, 0.5 mM EDTA, 0.5 mM DTT, 0.2 mM PMSF, 2 µg mL^−1^ PepA, 20 mM imidazole, 0.15% w/v DDM or 0.034% w/v t-PCCαM, pH 6.4 with KOH) and incubated with Ni-NTA resin (1 mL 50% v/v slurry per 40 mg protein, 16 h, 4 °C). The resin was washed sequentially with wash buffer containing 250 mM, then 50 mM KCl, and proteins were eluted with wash buffer containing 50 mM KCl and 500 mM imidazole. Eluates were concentrated to > 1 mg mL^−1^ using centrifugal concentrators with a cut-off at 50 or 100 kDa (Sartorius, Göttingen, Germany), buffer-exchanged into storage buffer (50 mM MES, 50 mM KCl, 20% v/v glycerol, 1 mM EDTA, 1 mM DTT, pH 6.4 with KOH) to dilute imidazole (< 2.5 mM), and either used immediately or flash-frozen at − 80 °C. Protein concentrations were determined at 280 nm using a NanoDrop spectrophotometer (Thermo Fisher Scientific).

### Size exclusion chromatography

SEC analysis was performed using a Superdex 200 Increase 10/300 GL column (GE Healthcare, Chicago, IL, USA) on an Agilent 1260 Infinity II LC system (Santa Clara, CA, USA) with 0.1 μm pore size inline filters (Wyatt Technology, Santa Barbara, CA, USA). The column was equilibrated with SEC-MALS buffer (50 mM 3-(*N*-morpholino)propanesulfonic acid, 150 mM KCl, 10% v/v glycerol, 1 mM EDTA, 1 mM DTT, 0.016–0.04% w/v DDM or 0.0036% w/v t-PCCαM, pH 7.5 with KOH). For activity assays on solubilized AHA2 oligomers in presence of lecithin, SEC was performed on an ÄKTA FPLC system (Cytiva). Depending on the downstream application, either up to 100 µg (size determination and assays on solubilized AHA2) or 720 µg (liposomal reconstitution) of AHA2 were loaded and eluted at a flowrate of 0.4 mL min^−1^. During oligomer separation for liposomal reconstitutions, the column was cooled to 10 °C with a Hitachi VWR ELITE LaChrom L-2350 column oven (VWR, Radnor, PA, USA). Fractions (250 µl) were used directly or snap-frozen and stored at − 80 °C. The absorbance signal at 280 nm recorded over time was extracted with the OpenLab software package, version c.01.08[210] (Agilent). Protein amounts in the fractions were calculated by converting the absorbance signal of the chromatogram into protein concentrations using the extinction coefficient and integrating the protein concentrations over the elution volume range for each fraction. Oligomer stability against high detergent concentrations was evaluated by increasing the DDM concentration from 0.04% w/v to 0.24% w/v before SEC. Protein samples supplemented with additional detergent were incubated for 30 min on ice prior to size exclusion chromatography. Dynamic re-equilibration of oligomeric states was investigated by re-loading isolated AHA2 monomer (3.6–3.9 µg, 8–11 µg mL^−1^) or trimer (3.5–25 µg, 14–67 µg mL^−1^) fractions under identical SEC conditions. Prior to size exclusion analysis, monomer and trimer fractions were either incubated on ice for 30 min or frozen in liquid nitrogen and stored at − 80 °C, then thawed on ice for 30 min.

For SEC-MALS experiments with a column of higher molecular weight exclusion limit, a Superose 6 Increase 3.2/300 column (Cytiva, Amersham, United Kingdom) was used. SEC-MALS analysis was performed with 50 µg AHA2 at a flowrate of 0.02 mL min^−1^.

### Oligomer size determination

The sizes of protein-detergent complexes in purified AHA2 samples were determined by SEC-MALS using a light scattering detector and a refractometer (TREOS II and Optilab T-rEX, Wyatt Technology) in-line with the same setup used for oligomer isolation. Acquired signals were pre-processed using ASTRA version 7.3.2.1 (Wyatt Technology) to adjust for band broadening and delay volumes between detectors. Molecular weights were calculated according to Slotboom et al.^46^. Membrane protein mass determination is complicated by the presence of detergent micelles, which create a heterogeneous protein-detergent complex with an often-unknown mass ratio. Because both components contribute to the absorbance and the refractive index, accurate evaluation of protein and complex masses requires accounting for their combined optical properties. As reflected in the extinction coefficient $\:{\epsilon\:}_{complex}$ and the concentration-dependent change of the refractive index $\:{\left(\frac{dn}{dc}\right)}_{complex}$ of the combined complex, the protein and detergent contribute to both coefficients based on their mass ratios. $\:{\left(\frac{dn}{dc}\right)}_{complex}$ can be calculated if the coefficients for the mass concentration-dependent refractive increment changes of the protein, $\:{\left(\frac{dn}{dc}\right)}_{prot}$, and of the detergent, $\:{\left(\frac{dn}{dc}\right)}_{det}$, are known:

1 $${\left(\frac{dn}{dc}\right)}_{complex}=\:\left(\frac{1}{1+\:\delta\:}\right)\:\cdot\:\:{\left(\frac{dn}{dc}\right)}_{prot}+\:\left(\frac{\delta\:}{1+\:\delta\:}\right)\:\cdot\:\:{\left(\frac{dn}{dc}\right)}_{det}.$$

The total mass of the complex is given as $\:1+\delta\:$, with $\:\frac{1}{1+\:\delta\:}$ representing the protein mass fraction and $\:\frac{\delta\:}{1+\:\delta\:}$ that of the detergent. $\:\delta\:$ can be resolved if the absorbance and refractive index of the complex have been measured. In our calculations, all equations assumed a pathlength of 1 cm and all spectral data was corrected for the buffer signal, resulting in the corrected refractive index $\:\varDelta\:RI$, measured at 658 nm wavelength, and the corrected absorbance at 280 nm $\:\varDelta\:{UV}_{280}$. At 280 nm, DDM does not absorb^46^, simplifying the equation to:

2 $$\delta\:=\:\frac{\frac{\varDelta\:RI}{\varDelta\:{UV}_{280}}\:\cdot\:\:{\epsilon\:}_{280,\:prot}\:-\:{\left(\frac{dn}{dc}\right)}_{prot}\:}{{\left(\frac{dn}{dc}\right)}_{det}}.$$

The extinction coefficient of AHA2 at 280 nm $\:{\epsilon\:}_{280,\:prot}$ was calculated as 1.113 mL mg^−1^ cm^−1^ based on the amino acid sequence using the ExPASy web application ProtParam^47^. An estimate for $\:{\left(\frac{dn}{dc}\right)}_{det}$ of DDM was taken from literature^48^. $\:{\left(\frac{dn}{dc}\right)}_{prot}\:$can be calculated based on the amino acid sequence of the protein^49^ as the amino acid composition is the deciding factor for most proteins^50–53^. However, as the buffer composition may alter $\:{\left(\frac{dn}{dc}\right)}_{prot}$^54^, $\:{\left(\frac{dn}{dc}\right)}_{prot}$ in the SEC-MALS buffer was approximated with an aldolase standard as described below and reported previously^46^, relying on the observation that $\:{\left(\frac{dn}{dc}\right)}_{prot}$ is similar for a wide range of proteins^49,52,55^.

Solving Eq. (2) allowed determination of $\:{\left(\frac{dn}{dc}\right)}_{complex}$ with Eq. (1). $\:\delta\:$ and $\:{\left(\frac{dn}{dc}\right)}_{complex}$ were calculated anew for each AHA2 chromatogram peak since each peak might signify a different association state. With a value for $\:{\left(\frac{dn}{dc}\right)}_{complex}$ and the light scattering signal $\:\varDelta\:LS$ measured at 661.6 nm, the molecular weight of the protein detergent complex $\:{M}_{w,complex}$ was assessed for each peak using the so-called two-detector approach:

3 $${M}_{w,complex}=\:\frac{\varDelta\:LS}{K{\left(\frac{dn}{dc}\right)}_{complex}\varDelta\:RI}.$$

The system-dependent constant * K* was determined through SEC-MALS analysis of aldolase (157 kDa,$\:\:{\epsilon\:}_{280}$ = 0.88 mL mg^−1^ cm^−1^) standards in SEC-MALS buffer. To do so, Eq. (3) was solved for * K*, with $\:\varDelta\:LS$ and $\:\varDelta\:RI$ from the aldolase standard analysis. $\:{\left(\frac{dn}{dc}\right)}_{complex}$ was substituted for $\:{\left(\frac{dn}{dc}\right)}_{protein}$ because aldolase is water-soluble without detergent micelles.

4 $$\:{\left(\frac{dn}{dc}\right)}_{prot}\:=\frac{\varDelta\:RI}{\left(\varDelta\:{UV}_{280}/{\epsilon\:}_{280,\:prot}\right)}.$$

The system constant * K* was then calculated according to Eq. (3) with the experimentally found $\:{\left(\frac{dn}{dc}\right)}_{prot}$. With these factors resolved, Eq. (3) was used to calculate the molecular weight of each AHA2-detergent complex. Separately for each peak, the molecular weight of AHA2 was retrieved by the three-detector method, which uses light scattering, absorbance and refraction data:

5 $$ M_{{w,\:protein}} = \frac{{\Delta LS \cdot \Delta UV_{{280}} }}{{K\left( {\Delta RI} \right)^{2} \varepsilon _{{280,\:protein}} }}. $$

Finally, the molecular weight of DDM in the complex was obtained as the difference of the protein-detergent complex size and the size of the AHA2 oligomer.

### Reconstitution into liposomes

Reconstitution of AHA2 oligomer fractions into liposomes followed the approach described in Lanfermeijer et al.^38^. Lecithin (L-α-phosphatidylcholine from soybean, Type II-S, 14–29% choline basis; Sigma-Aldrich) was suspended in reconstitution buffer (10 mM MES, 50 mM K_2_SO_4_, 20% v/v glycerol, pH 6.4 with KOH) to 30 mg mL^−1^ and destabilized by OG for 5 min at RT. Then, 12 µg of protein were added to a final volume of 220 µL of the reconstitution sample (10 mM MES, 50 mM K_2_SO_4_, 10.6 mg mL^−1^ lecithin, 52 mM OG, 5 µg/mL protein, pH 6.4 with KOH), which was incubated with end-over-end rotation for 5 min at RT. AHA2 monomer and dimer SEC fractions were selected according to the highest protein concentration within the respective SEC peak. For mock proteoliposome controls, an equivalent volume of SEC buffer was added instead of the protein. The detergent was removed by incubation on buffer-hydrated Sephadex G-50 resin for 5 min at RT and subsequent centrifugation at 180 × g, 8 min, RT, followed by incubation of the recovered eluate with 160 mg Bio-Beads for 30 min, RT. The resulting proteoliposomes were used immediately for activity assays and stored at − 20 °C for subsequent protein quantification.

To assess the oligomeric state of AHA2 after reconstitution, proteoliposomes (1 mL, 4.9 mg mL^−1^ lipids) were solubilized using a final concentration of 3% w/v DDM by incubation for 1 h at 4 °C. Insoluble material was removed by ultracentrifugation (100,000 × g, 1 h, 4 °C) and the solubilized AHA2 fraction was labelled with SNAP-Surface Alexa Fluor 647 (New England Biolabs, Ipswich, MA, USA) using a protein-to-dye molar ratio of 1:2 by incubating for 1 h at 4 °C. Solubilized and labelled protein was then analyzed with a Superdex 200 Increase 10/300 GL column, recording the Alexa Fluor 647 fluorescence (ex/em 652 nm/670 nm; Gain: 15) using an Agilent 1260 Infinity II high-sensitivity fluorescence detector. To represent the oligomeric state of AHA2 SEC fractions before reconstitution, enriched AHA2 monomer and dimer SEC fractions were labelled and analyzed under identical conditions.

### Activity assays

ATPase activity of detergent-solubilized AHA2 was quantified using a malachite green assay^56^. AHA2 (17–320 ng) was incubated at 30 °C for 30 min in ATPase assay buffer (105 µL; 20 mM MES, 8 mM MgSO_4_, 50 mM KNO_3_, 0.25 mM NaMoO_4_, 0.4 mg mL^− 1^ DOPC or lecithin, 0.05% w/v DDM, 2.9 mM ATP, pH 6.4 with KOH). Reactions were stopped with 800 µL malachite green stop solution (0.135 mM malachite green, 0.17% w/v polyvinyl alcohol, 0.95 M H_2_SO_4_, 7 mM ammonium heptamolybdate), followed by 100 µl 34% w/w tri-sodium citrate to halt color development. Liberated phosphate was quantified against inorganic phosphate standards via absorbance at 638 nm. ATPase activity of reconstituted AHA2 was quantified using an enzymatic NADH-coupled assay, linking ADP production to NADH oxidation^57^. ATP hydrolysis of proteoliposomes (15 µL) was measured in NADH assay buffer (20 mM MES, 52.5 mM K_2_SO_4_, 2 mM ATP, 2 mM phosphoenolpyruvate, 10 U mL^−1^ pyruvate kinase, 7 U mL^−1^ lactate dehydrogenase, 200 µM NADH, pH 6.4 with KOH) by initiating the reaction with 10 mM MgSO_4_. Specific ATPase activities were calculated from the linear decrease in absorbance at 340 nm using the NADH extinction coefficient (6.2 mM^−1^ cm^−1^), total volume, and AHA2 content. Background decreases from mock proteoliposomes were subtracted. Proton pumping was measured using the pH-sensitive fluorophore 9-amino-6-chloro-2-methoxyacridine (ACMA)^58^. Proteoliposomes (15 µL) were diluted with proton pumping buffer (20 mM MES, 52.5 mM K_2_SO_4_, 2 mM ATP, 62.5 nM valinomycin, 2 µM ACMA, pH 6.4 with KOH). Fluorescence (ex 412 nm/em 480 nm) was recorded over time, and pumping was triggered with 10 mM MgSO₄. Subsequently, 6 µM CCCP was added to collapse the proton gradient. Initial pumping velocities were normalized to the signal immediately after MgSO₄ addition and to the amount of reconstituted AHA2, with mock proteoliposome signals used for background correction. All measurements were conducted with a CLARIOstar Plus microplate reader (BMG Labtech, Ortenberg, Germany) in transparent 96-well microplates (Sarstedt).

## Supplementary Information

Below is the link to the electronic supplementary material.

Supplementary Material 1

Supplementary Material 2

## Data Availability

All datasets generated during this study are presented in this published article and the Supplementary Information files. For further inquiries, please contact the corresponding author.
