# Supplementary material for: Isolation and characterization of plant plasma membrane P-type H+-ATPase dimers
Source: Sci Rep. 2026 Sep 15;16:28775. doi: 10.1038/s41598-026-70970-z (PMC13578685; doi:10.1038/s41598-026-70970-z)
Supplement: Supplementary file 1 [file 41598_2026_70970_MOESM1_ESM.docx]

**Isolation and characterization of plant plasma membrane P-type H^+^‑ATPase dimers**

Ekaterina Malysenko^1^, Thomas Günther Pomorski^1,2^ and Bo Højen Justesen^1,^*

^1^Department of Molecular Biochemistry, Faculty of Chemistry and Biochemistry, Ruhr University Bochum, Bochum, 44780, Germany

^2^Department of Plant and Environmental Sciences, University of Copenhagen, Frederiksberg, 1871, Denmark

*corresponding author: bo.justesen@ruhr-uni-bochum.de

**Supporting Information**

*Supplementary data*

Figure S1. AHA2 purification.	S-2

Figure S2. AHA2 oligomer size determination.	S-3

Figure S3. Purity of AHA2 size exclusion fractions.	S-4

Figure S4. AHA2 oligomer detergent disruption test.	S-5

Figure S5. AHA2 monomer isolation.	S-6

Figure S6. AHA2 trimer isolation.	S-7

Figure S7. Detergent solubilization tests.	S-8

Figure S8. SDS-PAGE of reconstituted AHA2 proteoliposomes.	S-9

Figure S9. ATPase activity of AHA2 oligomers in presence of lecithin.	S-10

Figure S10. Oligomeric state of AHA2 after reconstitution into proteoliposomes.	S-11

*Full images and repetitions*

Figure S11. Repetitions of AHA2 purifications.	S-12

Figure S12. Full images of antibody binding tests.	S-13

Figure S13. Extended AHA2 size exclusion fraction analysis.	S-14

Figure S14. Full images of DDM solubilization tests.	S-15

Figure S15. Full images of t-PCCαM solubilization tests.	S-16

Figure S16. Full images of protein quantification in proteoliposomes.	S-17

**Figure S1. AHA2 purification. a** Yeast membranes diluted to 10 g L^-1^ total protein were solubilized (SL) with 0.5% w/v DDM. The solubilized fraction (S1) was separated from non-solubilized membranes (P) by ultracentrifugation and incubated with a nickel affinity resin for 16 h at 4°C. The unbound fraction (S2) was removed, and the matrix was washed twice with buffer (W1, W2). The eluate (E) was recovered from the matrix (C) and concentrated (E_c_) using a spin concentrator with a 100 kDa molecular weight cut-off. The concentrator flowthrough (FT) was also analyzed for the presence of AHA2. **b** Coomassie staining (COOM) and **c** western blots using an anti-RGS·His₄ antibody (α‑RGS·His_4_) revealed that approximately half of the total AHA2 was solubilized during detergent extraction. Essentially all solubilized AHA2 bound to the affinity resin and was released from the matrix during elution, as evidenced by a single band corresponding to the molecular weight of AHA2 (127 kDa, three independent purifications). **d** AHA2-containing membranes (three independent replicates) show a complex band pattern when analyzed by western blotting. **e** A similar pattern of secondary bands was observed in the negative control, in which the primary antibody incubation step was omitted and replaced with wash buffer alone, followed by incubation with the alkaline phosphatase-conjugated anti-mouse IgG secondary antibody. **f** Coomassie staining of membrane samples revealed a band pattern similar to that observed after incubation with secondary antibody alone (**e**), indicating that unspecific recognition by the secondary antibody was a major contributor to the detection of multiple bands. Black arrowheads mark the expected AHA2 band migration. Repetitions for experiments shown in **b-c** are included in Supplementary Figure S11. Full-image blots and gels for subfigures **b-f** are provided in Supplementary Figure S11‑12.

**Figure S2. AHA2 oligomer size determination**. **a** Two repetitions were performed of the experiment presented in Figure 1, obtained from biologically independent purifications (i, ii). After purification in presence of DDM, AHA2 separated into four peaks (P1-4) in size exclusion chromatography multi-angle light scattering (SEC-MALS) analysis using a Superdex 200 Increase 10/300 GL column (fractionation range: 10‑600 kDa). The sizes determined for the protein components (M_w, prot_), the detergent micelle (M_w, det_), and both protein and detergent micelle together (M_w, com_) are superimposed onto the corresponding elution peaks. The AHA2 monomer molecular weight corresponds to 127 kDa. **b** SEC-MALS analysis of total AHA2 was conducted using a size exclusion column with a higher molecular weight exclusion limit (Superose 6 Increase, 3.2/300, fractionation range: 5‑5,000 kDa). Because of its higher molecular weight exclusion limit, the Superose 6 column has a lower resolution in the range of AHA2 monomers, dimers, and trimers, which was evident from the elution of a single broad peak. Despite this limitation, the highest detectable AHA2 molecular weight (M_w, prot_) at the preceding edge of the peak corresponded to trimeric or tetrameric AHA2, whereas no hexamers were detected. Shown are two biologically independent purifications (i, ii).

**Figure S3. Purity of AHA2 size exclusion fractions. a** Size exclusion fractions of AHA2 were collected across all identified protein peaks (P1-4). The chromatogram corresponds to a single exemplary measurement (n = 4, independent purifications). **b** Visualized with Coomassie staining, electrophoretically separated size exclusion fractions showed AHA2 (black arrowhead, 127 kDa) as the only detectable protein. Due to the application of equal fraction volumes, the band intensity was proportional to the AHA2 concentration in all fractions. A full gel image and independent repetitions are provided in Supplementary Fig. S13.

**Figure S4. AHA2 oligomer detergent disruption test.** AHA2 was mixed into sample buffer with 0.24% w/v DDM and incubated for 30 min on ice to probe oligomer disruption through increased detergent concentrations. Detergent-treated AHA2 was then analyzed by size exclusion chromatography analysis and compared to AHA2 in regular storage buffer containing 0.04% w/v DDM as a control (n = 2, independent purifications). Monomer peaks were unaffected by detergent incubation, indicating no disruption of oligomers.

**Figure S5. AHA2 monomer isolation. a** Large amounts of AHA2 (i, 680 µg; ii, 695 µg) were subjected to size exclusion chromatography. A fraction from the monomer peak was collected, incubated on ice for 30 min, and reapplied to the same size exclusion column. **b** Re-chromatography of the isolated monomer-enriched fraction resulted in improved separation between AHA2 monomer and dimer species. To further increase monomer purity, another monomer-enriched fraction was recovered, incubated on ice for 30 min, and reapplied to the column. **c** Sequential isolation of monomer-enriched fractions resulted in a mostly isolated AHA2 monomer peak. The elution ranges of fractions recovered for reapplication are indicated by black arrow heads. Two independent purifications are shown (i, ii).

**Figure S6. AHA2 trimer isolation. a** To examine dynamic redistribution of AHA2 oligomers, total AHA2 (i, 570 µg; ii, 598 µg) was separated by size exclusion chromatography. A fraction of the trimer peak was collected (volume range indicated by black arrowhead). **b** The collected, trimer-enriched fraction was incubated on ice for 30 min and subsequently reapplied to the size exclusion column. The resulting chromatogram revealed a single peak within the elution range of the reapplied trimer fractions. Two independent purifications are shown (i, ii).

**Figure S7. Detergent solubilization tests.** Solubilization efficiency of AHA2 from yeast membranes was tested using the detergents DDM and t-PCCαM. After solubilization, soluble and insoluble fractions were separated via ultracentrifugation. The resulting supernatants and resuspended pellets were analyzed via SDS-PAGE followed by western blotting. The solubilization efficiency was calculated by comparing the band intensity of AHA2 in the supernatant to the total intensity (supernatant + pellet). **a-d** For DDM, AHA2 solubilization reached a plateau of approximately 50% at a concentration of 0.5% w/v DDM added to 10 mg mL^‑1^ membrane protein. **e-h** The same solubilization efficiency was observed for t‑PCCαM. DDM-solubilized samples of the same membranes, analyzed on the same blot, confirmed similar solubilization behaviors of the two detergents. Full blots are shown in Supplementary Fig. S14-15.

**Figure S8. SDS-PAGE of reconstituted AHA2 proteoliposomes.** AHA2 monomers (mono) and dimers (di) from separate purifications (set 1-3) were reconstituted into lecithin vesicles in technical replicates (**a-b** set 1 and 2, **c** set 3). Additional reconstitutions included AHA2 before size exclusion chromatography (total) and mock liposomes without protein. AHA2 amounts in proteoliposomes (black arrowhead) were estimated from a BSA standard series (gray arrowhead) after Coomassie staining. L marks the protein ladder lane. Full gel images are shown in Supplementary Fig. S16.

**Figure S9. ATPase activity of AHA2 oligomers in presence of lecithin.** ATPase activity of DDM-solubilized AHA2 monomers (mono) and dimers (di) was measured in the presence of 0.4 g/L lecithin (n = 2).

**Figure S10. Oligomeric state of AHA2 after reconstitution into proteoliposomes. a** Size exclusion chromatography (SEC) fractions enriched in AHA2 dimers (F8 and F9) and AHA2 monomers (F13 and F14) were reconstituted into proteoliposomes. **b** The oligomeric state of AHA2 was analyzed by SEC before and after reconstitution into proteoliposomes and subsequent solubilization with DDM. AHA2 was fluorescently labelled via the N-terminal SNAP-tag for detection (LU, luminescence units). All samples contained heterogeneous populations of AHA2 assembly states, resulting from limited separation in the initial sample preparation. Based on differences in peak fluorescence intensities, qualitatively, monomer-enriched SEC fractions displayed a greater monomer/dimer ratio than dimer-enriched fractions. After reconstitution and solubilization, overall, AHA2 extracts from monomer proteoliposomes maintained a monomer/dimer ratio favoring monomers, while dimer proteoliposomes displayed a lower monomer/dimer ratio, exhibiting greater dimer than monomer peak intensities. The elution ranges for AHA2 monomers (mono), dimers (di), trimers (tri, presumed assembly state), and aggregates (agg) are indicated

**Figure S11. Repetitions of AHA2 purifications. a** Outlines of cropped images used in Supplementary Figure S1**b-c** were marked in gray. **b-c** Additionally, two independent repetitions of the experiments shown in Supplementary Figure S1b-c are depicted. L, protein ladder.

**Figure S12. Full images of antibody binding tests.** Gray boxes mark the cropped regions used in Supplementary Fig. S1**d-f**. L, protein ladder.

**Figure S13. Extended AHA2 size exclusion fraction analysis. a** An expansion of the chromatogram depicted in Supplementary Fig. S3 included an additional micelle peak, which contained no detectable proteins (**b**). **b** A gray box marks the cropped region used in Supplementary Fig. S3. **c-f** Independent repetitions of **a-b**. L, protein ladder.

**Figure S14. Full images of DDM solubilization tests. a-b** Gray boxes indicate cropped regions used in Supplementary Fig. S7. L, protein ladder.

**Figure S15. Full images of t-PCCαM solubilization tests. a-b** Gray boxes indicate cropped regions used in Supplementary Fig. S7. L, protein ladder.

**Figure S16. Full images of protein quantification in proteoliposomes. a-c** Gray boxes indicate cropped regions used in Supplementary Fig. S8. L, protein ladder.
