# Supplementary material for: Isolation and characterization of plant plasma membrane P-type H+-ATPase dimers
Source: Sci Rep. 2026 Sep 15;16:28775. doi: 10.1038/s41598-026-70970-z (PMC13578685; doi:10.1038/s41598-026-70970-z)
Supplement: Supplementary file 2 [file 41598_2026_70970_MOESM2_ESM.docx]

**Supplementary Information**

Supplementary information is available for download.

Supplementary Fig. S1 shows SDS-PAGE analysis of AHA2 purification steps. Biologically independent replicates of SEC-MALS measurements are shown in Supplementary Fig. S2. Supplementary Fig. S3 displays AHA2 sample purity in SEC fractions. Oligomer stability testing results are depicted in Supplementary Fig. S4-6. Detergent solubilization tests are shown in Supplementary Fig. S7. Supplementary Fig. S8 shows gels used for quantification of AHA2 in proteoliposomes. ATPase activity assays of AHA2 in presence of DDM and lecithin are depicted in Supplementary Fig. S9. Supplementary Fig. S10 demonstrates the recovery of distinctly different AHA2 oligomer states in proteoliposomes after AHA2 dimer and monomer reconstitution. Supplementary Fig. S11-16 contain repetitions and full images of data shown in the Supplementary Information.
